# A Study on the Improvement of College Students’ Psychological Pressure and Anxiety by Using English Psychological Script Activities

**DOI:** 10.3389/fpsyg.2022.878479

**Published:** 2022-04-27

**Authors:** Lina Mu, Baiyan Du, Xuemei Hou

**Affiliations:** ^1^School of Foreign Languages and Literature, Tianjin University, Tianjin, China; ^2^Department of Educational Science, Sehan University, Yeongam, South Korea

**Keywords:** psychological pressure, anxiety, mental health improvement, English psychological, script activity

## Abstract

This study conducted an experiment of English script activities on 279 senior students from two universities in Guangdong Province, China. The purpose of this study was to explore the effect of English psychological script activities on improving the psychological pressure and anxiety of college students. The results show that, firstly, before the experiment, the overall psychological pressure and anxiety of college students are at a medium high level. The gender difference shows that the psychological pressure and anxiety level of girls are higher than that of boys. The professional difference shows that the psychological pressure and anxiety level of Humanities and social sciences majors are higher than that of science and engineering majors. After the experiment, the overall psychological pressure and anxiety level of college students have a significant improvement effect. From the overall level, English psychological script has the highest impact on evaluation anxiety and test anxiety. From the perspective of gender differences, English psychological scripts have the highest effect on improving the evaluation anxiety of boys, and the effect of improving test anxiety and evaluation anxiety of girls is the highest. From the perspective of professional differences, English psychological scripts have an average impact on the psychological pressure and anxiety of students majoring in Humanities and Social Sciences, while they have the highest impact on the interpersonal stress of students majoring in science and technology. The results of this research provide more reference value for college students’ English education and mental health improvement.

## Introduction

With the development of society, the pressures and anxieties people encounter in life are becoming more frequent and diversified. Pressure and anxiety are non-specific reactions that may occur in the human body under any conditions (Fink, 2017). They are psychological contradictory stimuli brought to individuals in the interaction between individuals and the environment, such as reactions to diseases, troubles in daily life, and disasters. They are also often expressed as negative factors when they cannot adapt to the requirements of the environment. Individuals will experience stress and anxiety when their requirements are frustrated by the environment and unable to respond appropriately, but if the pressure and anxiety are at an appropriate level, it will be helpful for personal growth. But despite this, everyone has different levels of stability for pressure and anxiety, and pressure and anxiety are both cumulative or sudden. Such anxiety and pressure will threaten the individual’s stable state. Therefore, if you are exposed to excessive levels of sudden stress and anxiety, as well as long-term excessive pressure and anxiety, it will be harmful to the individual. According to the research of Tcha (2020) and Aida (1994), the prevalence of anxiety and pressure at the social level in Korea increased three times in 2016 and 2011, and the psychological prevalence rate is the highest in the age group of 18–29-years-old (Fan and Li, 2000; Dimsdale, 2008; Aktekin et al., 2019; Coffman and Gilligan, 2020; Jain et al., 2022). Among them, the anxiety and pressure of college students are most likely to be affected by the social situation. The prevalence of pressure and anxiety is 10% in the United States and 2.4% in Europe (Firth, 1989). The stress and anxiety of Chinese students are mainly reflected in the three fields of study, life, and employment. According to a study by Hua (2017) of 608 students in the upper grades of primary schools, the results show that 40% of primary school students have high stress and anxiety states since the upper grades, which are closely related to exams. The research of Lin et al. (2005) shows that academic pressure accounts for 42.3%, employment 22%, and life 10% among students. It can be seen that in the current fast-paced life, stress and anxiety are gradually getting younger, and show the same growth trend with the development of society.

For college students, the huge amount of discipline learning and employment situation are asymmetric, which undoubtedly causes more academic pressure on contemporary college students. In addition, if college students lack their own learning planning and future development goals in the early stage of entering the school, they may suddenly face serious anxiety and stress in the senior year or after graduation. The internal competition of these majors also reflects a trend of academic pressure on academic achievement, which is not conducive to the growth of individuals in the social environment. According to the research of Zhong et al. (2018), there were nearly 7.49 million college graduates nationwide in 2018 (Gall et al., 2000; Goldman and Delcy, 2003; Hamaideh, 2011; Liu and Liu, 2021). The employment situation is relatively severe, and the employment pressure is increasing year by year, mainly from the social and family environment, personal self-evaluation, employment expectation, and so on. From the perspective of school life, college students will encounter frequent academic tasks, economic pressure and interpersonal pressure in their study and life. The long-term accumulation of these pressures leads to the continuous increase of students’ anxiety experience time. College students with ordinary grades may also face the problems of negative factors such as lack of self-confidence, habitual avoidance, and inferiority. A study by Hamaideh (2011) pointed out that the total amount of stress and anxiety felt by students worldwide increased to 16% in 1985 and 27% in 2002 (Lazarus and Folkman, 1984; Lang et al., 2000; Hu et al., 2013; Himle et al., 2014; Kong et al., 2020), among which 75–80% felt moderately and 10–12% felt too much stress and anxiety. In the rising pressure and anxiety of college students, the five most important factors are learning, economy, time management, interpersonal contradictions, and internal psychological difficulties. Among them, the factors related to learning activities have a great impact on students’ stress and negative emotional state, and these factors make students’ negative state higher than that of other stages. It can be seen that college students are a long-term experience group of stress and anxiety.

In order to improve the situation of stress and anxiety among college students, different researchers have taken measures to solve this problem. Ham (2018) implemented special dance activities to reduce students’ anxiety and stress. Afterwards, it was found that the stress level decreased compared with the first test, especially in the factors of interpersonal relationship and values (Lewis, 1970). Kim (2015) conducted 18 collective art therapy activities for college athletes. The results show that art therapy can effectively reduce the stress and depression of college athletes (Lumsden, 1981; Lv and Zhu, 2005). Chen (2019) applied psychodrama in mental health education and teaching, through the campus psychological script performance close to students’ life, let students experience psychological confusion and cognitive conflict, and guide students to actively think about the problems they encounter and explore solutions. Kipper (1978) used psychodrama to study violent crime and social and family problems, and integrated psychodrama with structured family counseling. Liu (2014) studied the effect of English psychodrama therapy on stress and found that English psychodrama therapy can not only effectively alleviate negative emotional states, but also improve language learning ability and interest (Liu, 2007). The age range of the treated population is also broad. Therefore, the methods to improve stress and anxiety are gradually enriched. From the perspective of language function, it is a cognitive ability of human beings, which can intuitively reflect individual emotions and psychological feelings. Therefore, individual anxiety and stress can be effectively, quickly, and intuitively perceived in language organization. When the human brain is in the state of organic input of the target language, if the individual’s anxiety and pressure are too high, the brain cannot be absorbed and transformed, and output disorders will be formed, mainly in the form of lack of self-confidence, depression, fear of negative evaluation, and so on. In the case of using a second language as a non-native language, since the individual does not have the same habit and affinity for the second language as the native language, the arousal degree of anxiety and stress characteristics of individuals will be more significant, and the intervention and treatment direction will become more clear (Kellermann, 1992; Liu, 2014, 2016). Wu (2020)‘s research suggests that the changes in students’ behavior and the improvement of language ability can be effectively seen in the process of English script activities, and it can also enhance students’ planning ability (Kim, 2015). You (2018) conducted a study on English psychological script activities for senior high school students (Moreno, 1953). The results showed that psychological script can release emotions, alleviate anxiety in English learning and improve learning pressure. SerLife (2010) conducted an English curriculum combined with psychodrama on teenagers, the results show that teenagers not only learn a large number of new vocabulary while performing psychodrama activities, but also have sufficient experience in intelligence and emotion, therefore, these students not only do not have to recite words alone, but also interact with other students to express their stress and anxiety. Mine (2015) took senior high school students as the object to study the learners’ anxiety about English learning. After pre- and post-test, it was determined that English psychodrama activity can effectively reduce learners’ English-speaking anxiety.

Based on the above research background, we know that psychological script activities often intervene and improve in the form of second language. English script activities can not only promote the interest in foreign language learning, but also positively affect the individual’s psychological status, and make effective use of the combination of language text and high-quality content. The function behind it can make individuals have a better effect on improving their self-condition. However, most of the English psychological script activity therapy mainly focuses on the observation of the improvement of English learning interest and language ability, while the research on the improvement of psychological pressure and anxiety after psychological script activity is still insufficient. The purpose of this study is to use English script activities to improve college students’ academic pressure, interpersonal pressure, self-future development pressure, social anxiety, evaluation anxiety, and test anxiety. This research believes that the existing research on psychological scripting activities has proved its effect on improving anxiety and pressure. If the psychological script activity is carried out in the second language situation, the attributes of language logic achievement and language learning achievement can be used to further improve the individual’s anxiety and stress state. Therefore, this study puts forward the following two research questions and three hypotheses:

First, what is the effect of psychological script activity on college students’ psychological stress and anxiety before and after the experiment? The research hypothesis of this part is H1. There are significant differences in the psychological stress and anxiety of college students before and after the psychological script activity experiment. Second, what are the effects of psychological script activities on gender and professional categories? The research hypothesis of this research problem is the following two points: H2 There are significant differences in psychological stress and anxiety between male and female students before and after the experiment. Last, H3 there are significant differences in psychological stress and anxiety between science and engineering majors, and humanities and social sciences majors before and after the experiment.

## Research Background

### Psychological Script and Mental Health

Psychological script is a treatment method for many aspects of negative factors. It is of great significance to sort out self-inner theory under the mode of theoretical activities and practical guidance. It was originally proposed by Moreno (1953) and Nelson et al. (2000). At first, it was used to explore how to use stage performance to develop people’s spontaneity and creativity in different environments. Later, it was gradually found that the traditional stage performance was constrained by the predetermined plot and fixed lines, and the performers could not play independently, resulting in the fact that stage performers and writers could not achieve a high degree of unity in their creations. Therefore, he began the experiment of self-drama performance in cooperation with some like-minded actors. In the process of self-drama performance, there are many components of impromptu performance, and the performer can always combine with his own experience in all aspects of life. In the impromptu performance, the psychological condition of the performers has also been greatly improved. Especially at the end of the performance, the performers always have the emotional condition of release and expression, which makes Moreno dig out that the psychological drama has a certain self-healing function. Then he began to enrich his practical experience in drama and integrate a set of social and psychometric treatment schemes, mainly in the form of actors following the suggestions of the live audience and the coordination of the director to perform creative drama. Over a long period of development, psychodrama has formed an important therapeutic genre with the joint efforts of various fields. Among them, 28 regions have established relevant organizations and institutions, including the Beacon Morey Institute in New York, and the Howell Psychological Script International Training Center in the United Kingdom. After many practical activities under the development mode of psychodrama, psychodrama has been proved to be effective in the clinical and consulting fields. Different from ordinary drama, psychodrama refers to re-experiencing our respective lives. It has the characteristics of spontaneity, creativity, flexibility, and the ability of self-expression. It not only provides opportunities for emotional catharsis and psychological rehabilitation, but also provides a re-examination and evaluation of inner confusion and conflict. Drama is mainly based on limited scripts and roles, and serves the stories of others. Therefore, drama serves the audience, while psychodrama serves people themselves. In some previous studies, the treatment research of psychodrama can be aimed at different groups, especially those with high level of fear and timidity. It is a practical plan to solve problems, It also has a good effect on special events (Williams and Robert, 1997; Park and Kim, 1999). Kellermann (1992) carried out psychological script treatment on patients with mental diseases and found that interpersonal relationship and emotional catharsis are important factors in psychological script group treatment (Robinson et al., 2011). At the same time, he pointed out that this can also play a good role in alleviating the conflicts and contradictions of teenagers and alleviating school violence.

The introduction of psychodrama into China began in the 1980s. After scholars in the field of psychology and education gradually understood its content, they began to practice it, and it is widely used in family guidance, unit staff training, psychotherapy professional work, language teaching, etc. Especially in language teaching, the combination of psychodrama is widely used, which can not only impart knowledge, but also play a role in mental health treatment. Hu et al. (2013) divided the experimental subjects into two groups (Schlenker and Leary, 1982). One group was treated with medication and the other group was treated with psychological script therapy. A total of four phases of observational studies were carried out. The results showed that the patients receiving psychological script therapy had a good therapeutic effect on depression and anxiety, and their self-confidence improved significantly. Zhou and Tang (2002) found that if psychological script therapy is integrated with mental health education, coupled with local elements in various places, it can improve interpersonal communication, reduce their own depression, anxiety, stress, and other problems, promote individual development and increase the rate of positive emotional experience (Selye, 1936, 1956)^.^

Through some previous studies, it can be seen that psychological script activities have a positive impact on mental health, improve the negative inner characteristics of different groups and delay the occurrence of mental diseases. From the perspective of psychodrama therapy technology, the reason why people often have different degrees of psychological problems is that desire is suppressed in the subconscious, and the self-defense system in the human body will prevent the intervention of subconscious content, so participants have more difficult self-identification of their own problems. However, in order to successfully involve the subconscious of the participants, psychodrama arranges safety and comfort factors in the links of the activities, so as to reduce the self-defense of the participants, reasonably vent the suppressed negative psychological emotions, and improve the mental health level of the participants. The key is to make participants integrate themselves psychologically by means of catharsis, that is, to re-examine the blind spots of themselves in all aspects of life and improve their cognition. For this reason, there are three stages in the treatment process of psychological script, whether individual or group. In the initial stage, members can form a solid cooperative trust among themselves and select roles in the script. Each role has different functions, which can be emotional or cognitive, however, the unified function is to help members integrate into the role to develop the promotion of the script, strengthen members’ sense of cooperation and express their inner world. In the mid-stage, after the members determine the role, the problem is brought from the surface to the core itself, and the scene is selected for the performance. The script also has a wide degree of freedom and is guided by the director. During the performance, each character needs to directly reveal the elements such as events and feelings, focus on the transformation process of emotional attitude, break the habitual thinking with the combination of sensibility and rationality, and understand the dilemma with new thinking. In the final stage, individuals and directors share with each other, reflect on themselves, experience the feelings of others and apply them to real life experience, while members cannot evaluate each other and can only listen and think. Finally, the director evaluates the results according to the depth of script analysis (Sherina et al., 2003; Liu, 2014). Therefore, it can be said that the activities of psychological script involve the responsibility and ability to the research object, director, therapist, and group leader. To complete the above three stages, various links are required, such as measurement, scene reproduction, role transformation and observation, inner transformation, value selection and behavior expression, the process of input to output is completed step by step from shallow to deep. Measurement refers to the construction mode between teams, which is mainly used to measure the relationship between members in the form of members arranged in a straight line and free movement after the initial stage. When the members are arranged in a straight line, there are two extreme emotional factors at both ends of the straight line. The members are arranged, and the middle is divided into different levels. The members can choose a certain position to represent their current state for expression. The form of free activity is to make members stop suddenly while walking at will, and then move briefly to determine their more comfortable position, so as to understand the acceptance of members to the group and observe the impact of member relations. The reappearance of the situation is described according to the development of the plot and the needs of the protagonist, and the situation is set according to the social life theme of common concern of the group. The role transformation and observation is that the performer replaces the original role with another role, for example, the supporting actor temporarily plays the protagonist, while the protagonist plays other characters. At this time, the person who replaces the role will observe his original role from the perspective of bystanders, help each role have a new understanding of the situation and produce a vivid feedback response (Tcha, 2020). Inner conversion and value selection are for the protagonist to explain and perform his inner two sides, such as victory and defeat, goodness and evil, reflect the sense of conflict, and recognize and accept the reality of this conflict. Behavior expression is the role represented by group members to express their relationship with others with the help of position and body language.

### Factors That Induce Psychological Pressure on College Students

Pressure is a necessary condition for modern people to survive. Its concept and definition has a long history. It was used in real life as early as the 14th century. It was mostly related to suffering, adversity, and difficulty (Lumsden, 1981), and then its content becomes richer, such as external factors that have adverse effects and cannot be resisted, or forced or tense state. Up to now, the definition of pressure has become more and more rich. Lazarus and Folkman defined pressure as external and internal stimuli perceived by individuals (Woo, 2016; Wu, 2020). These stimuli will stimulate emotions and even threaten health and life. People need to adapt to these stimulating changes and physiological and behavioral responses. In the relationship between individuals and the environment, the resources to deal with pressure exceed the scope of individual tolerance, making people feel a certain threat. However, the pressure will be different due to different perspectives. It can be divided into adaptive pressure, stimulating pressure, and interactive pressure. Adaptive pressure is the response to psychological or physiological changes trying to adjust the balance, that is, it is the response to various negative stimuli trying to adjust and adapt. According to Selye, adaptive pressure is that when an event changes, the body will use all resources to take internal response measures. Stimulating pressure regards pressure as a factor that can affect the outcome of the event, that is, the generation of pressure may be a challenge or change to the event. Interactive pressure emphasizes the process of individual and environmental changes, emphasizing the stimulus factors in the environment and the individual’s cognition and coping ability (Yao, 2020).

There are many factors that cause pressure, whether in the fields of physics and chemistry or psychology and society, they will cause pressure factors, and the degree of pressure and the cumulative amount of time are also different. From the perspective of individual analysis, usually when an individual is negatively evaluated without being recognized by others, when he is aware of his own incompetence, when he feels empty mentally, when he is physically embarrassed, etc., these situations will lead to varying degrees of pressure. The anger generated by pressure accumulation is aggressive and fearful. Long-term pressure can reduce self-immune function, produce fatigue, reduce psychological defense ability, and constantly produce self-inner contradictions, leading to mania, paranoia, or depression. These mental illnesses are mostly characterized by sadness and loss, and are also accompanied by emotional disorders such as persistent loneliness, accusations, feelings of guilt, and lack of interest and happiness. In thinking, it is easy to lose concentration, cognitive distortion, insomnia, loss of appetite and other physiological symptoms. From the perspective of environment, natural disasters will bring huge pressure stimulation to local ecology and society. If accumulated for too long, it will lead to continuous accident damage, and this huge external pressure stimulation will be transmitted and distributed to each unit or individual level by level from top to bottom. Therefore, cumulative pressure is not a problem that can be despised.

Among college students, due to the continuous competition in society, the tense employment atmosphere, academic examinations and other related factors, the pressure they experience is also increasing (Xu and Zhong, 2002). Coffman and Gilligan (2020) pointed out that most of the stressors come from time management, managing diverse interpersonal relationships, worrying about employment, prospects and academics, etc. Among these pressure sources, the pressure of future and self-development has the attribute of long-term accumulation of pressure, which is easy to accompany the entire experience of college students in the student period. Therefore, stress can reflect the indicators of college students’ living conditions, self-evaluation, and psychological tolerance. According to the data survey of Tsinghua University in China by Fan and Li (2000), 71.3% of college students are suffering from great psychological pressure and further physical and psychological adverse reactions (Zhou and Tang, 2002). These pressures come not only from academic fields, but also from employment, life, economy, growth and other fields, and the comprehensive degree of pressure is also showing an upward trend. Pierceall et al. conducted a survey of 212 college students in the United States, and found that 75% of college students are at a moderate pressure level, 12% of college students are at a high pressure level, and 13% of college students are at a low pressure level. In the gender level difference, the research of Che et al. (2003) shows that female college students have higher pressure on job hunting and academic work than boys, and male college students have greater pressure on themselves. This difference is related to social expectations. Xu and Zhong found in the study of college students in Zhejiang province that the pressure of male students in learning and social life is higher than that of female students. In the study of Aktekin et al. and Sherina et al. on students’ stress, it is shown that the pressure of girls is higher than that of boys. Among the differences in grade level, the research results of Guo (2007) show that the higher the grade, the higher the psychological pressure of college students, which gradually shows an increasing trend (Lang et al., 2000). The research of Firth shows that the stress of senior college students is higher than that of freshmen and sophomores. The stress of senior and junior students is the peak period of college students. Among the differences in the level of pressure among students of different majors, Guo said that the sense of psychological pressure of students majoring in liberal arts was significantly higher than that of students majoring in science. Liu (2007) found that college students majoring in literature, history and art scored higher in self-development, life and job selection than college students majoring in other majors (Lewis, 1970).

Through the above research, it is found that stress is an increasingly prominent problem of senior college students, which is in the middle and upper trend in most surveys, and the problems in study, interpersonal relationship and employment are often in the most prominent position. Although there are different effects according to different demographic characteristics, it can be seen from the overall level that with the development of society, this has become an important factor affecting their physical and mental health. The college stage is a period of rapid physical and psychological development. Appropriate pressure can have a positive impact on the individual. However, when senior students gradually transition to the social environment, the pressure may increase, which will also affect the stability and variability of students. Therefore, this study sets the research object as senior students, and considers the psychological stress component as the three aspects of the students’ academic pressure in university life, the pressure of interpersonal relationship and the pressure of self-development.

### Relationship Between Anxiety and Psychological Pressure

Anxiety is an emotional state that humans often encounter in life. According to a survey by Robinson et al. (2011), more than 25% of people will experience frequent anxiety during their lifetime, and it will be accompanied by a series of chain negative effects, such as economic or emotional loss. Therefore, the definition of anxiety is always accompanied by subjective feelings of tension, worry and fear, which is a common symptom to unknown results of events. At the same time, individuals will also experience the state of psychological pressure when they feel anxiety. Since the application of anxiety theory in research, different fields and scholars have different definitions of anxiety, so it is impossible to form a unified definition and explanation. In psychoanalytic theory, anxiety is the nervous system symptom caused by the failure of individual libido release. Behaviorist theory believes that anxiety is the self-defense state of individuals in the face of external environmental threats, which promotes stimulating behavior to form new habits. Cognitive theory believes that anxiety is an individual’s misattribution of events, and it is also the result of irrational beliefs (Ellis, 1977). Humanism believes that anxiety is an inevitable emotional experience of individuals in the world. It is often associated with the emotion of fear. In addition, for different leading researchers, Lang et al. (2000) believes that anxiety is the physiological reaction or psychological emotion of anxiety experienced when the individual consciousness is threatened by the upcoming event, and it will cause cognitive stress (Lv and Zhu, 2005). Lewis (1970) defined anxiety as an unpleasant and negative emotional experience process. In some previous studies, anxiety was also classified, which can be divided into state anxiety and trait anxiety. When individuals are stimulated, they will evaluate themselves. If the stimulation is threatening, they have the conditions of state anxiety, such as on-the-spot performance. Therefore, state anxiety is a temporary and scene-oriented factor. Trait anxiety is defined as a long-term and stable factor, which is caused by individual differences in personality and will not change due to time and place (Dimsdale, 2008). According to the two factors of state anxiety and trait anxiety, some researchers have divided them into social, academic, and other anxiety factors. Social anxiety is a phenomenon that individuals feel anxious when they are evaluated in social situations. Social anxiety occurs frequently in adulthood. Social anxiety in childhood and adolescence is mainly caused by family environment (Nelson et al., 2000). Academic anxiety is a series of negative reactions that students experience in the school environment with examination and learning as the main factors (Schlenker and Leary, 1982). Therefore, although the individualization of anxiety is different, in general, anxiety is mainly focused on the relationship between individual response and external environment.

The anxiety of college students mainly involves examinations, social interactions and other fields, and the university is in a stage where people are gradually moving towards the maturity of mental development. They have a subjective two-level tendency of success or failure in the event they face, forcing individuals to have more or less adaptive barriers and adverse emotional reactions to different fields. Lv and Zhu (2005) conducted a survey on the anxiety status of college students (Kim, 2015). The results show that 38% of college students are deeply troubled by anxiety, and the level of anxiety is different according to their grade and family status. Moreover, boys are more concerned about their academic life and interpersonal relationship than girls. Yao (2020) believes that groups with high anxiety and pressure will be accompanied by high depression (Lumsden, 1981). The anxiety of college students mainly includes immediate stress and tension. If students’ confidence is relieved and stress response measures are taken, the level of anxiety can be alleviated. Himle et al. believed that college students’ anxiety disorder has a negative impact on psychological pressure, which is due to the lack of corresponding response measures. For example, when students lack the required interview skills before employment, anxiety and stress will be activated at the same time. If they are completely unprepared for some exams and emergencies, anxiety and stress will have a negative impact on individual execution and self-confidence, increasing their sense of self-doubt.

It can be seen that anxiety and pressure are both negative factors for individual psychology. The inducing conditions of the two factors are not carried out separately, but factors that occur simultaneously and may have a long-term nature. The anxiety in this study refers to three factors: social anxiety, evaluation anxiety, and examination anxiety among college students. Combining the three parts of psychodrama, pressure, and anxiety, the activities of psychodrama include six parts: team and reunion, rejection of fear, giving self-confidence, self-challenge, release and evaluation, and goals and planning. According to these sections, the stress and anxiety of this study also have six sections: academic pressure, interpersonal pressure, self-development pressure, social anxiety, evaluation anxiety, and examination anxiety. Team reunion and self-challenge activity links are aimed at interpersonal stress and social anxiety. The activity links of rejection of fear, release and evaluation are aimed at evaluation anxiety and examination anxiety. Giving self-confidence, goals and planning links are aimed at academic pressure and self-development pressure. Therefore, these links solve students’ individual development, guide mental health, increase positive emotional experience, and help students understand psychological problems.

## Research Methods and Results

### Research Objects

The objects of this study are senior students from two universities in Guangdong Province, China. A total of 316 students participated in the experiment before the experiment. Finally, a total of 279 students are effective samples, which are the key research objects of this study. Table 1: summarizes the distribution of objects used in the final analysis, including 142 girls and 137 boys. According to discipline classification, there are 114 science and engineering majors, and 165 humanities and social sciences majors.

**Table 1:** Research objects.

Category		Number of people
Gender	Boy	137
	Girl	142
Professional category	Science and engineering	114
	Humanities and social sciences	165
Total		279

### Anxiety and Psychological Pressure Measurement Tool

The anxiety scale used in this study adopts the anxiety questionnaire used by Liu (2014), which is developed by Horwitz et al. (1986), with a total of 38 questions and an overall reliability of 0.801, of which 1–10 questions are social anxiety, the reliability is 0.742, 11–20 questions are evaluation anxiety, the reliability is 0.735, 21–38 questions are examination anxiety, and the reliability is 0.830. The psychological pressure measurement tool adopts the pressure questionnaire developed by Park and Kim (1999), with a total of 15 questions, and the overall reliability is 0.796, of which 1–5 are academic pressure, the reliability is 0.814, 6–10 are interpersonal pressure, the reliability is 0.736, and 11–15 are self-development pressure, the reliability is 0.711. The 5-point Likert scoring method of “strongly agree (5 points), agree (4 points), general (3 points), disagree (2 points) and completely disagree (1 point)” is both adopted. Table 2: shows the problem composition and reliability of anxiety and psychological pressure.

**Table 2:** Question composition and reliability of anxiety and psychological pressure questionnaire.

Variable	Item		Reliability
Social anxiety	1–10	10	0.742
Evaluation anxiety	11–20	10	0.735
Examination anxiety	21–38	18	0.830
Anxiety	26	0.801	
Academic pressure	1–5	5	0.814
Interpersonal pressure	6–10	5	0.736
Self-development pressure	11–15	5	0.711
Psychological pressure	15	0.796	

### Research Procedures

In this study, SPSS 25.0 software was used to test the data results, and the reliability of the questionnaire was verified by Cronbach’s *α* coefficient. The experiment took 1 week in total. Before the experiment, the scores are recorded, and then the English psychological script activity is implemented. Before the activity, the English text and content will be explained and trained, and preparation time will be given. The theme and activity outline of the experiment are shown in Table 3. After the psychological script activity, the evaluation was carried out again to compare the differences before and after the experiment.

**Table 3:** Activity summary and cycle.

No.	Theme	Activity outline	Cycle	Time plan
1	Team and reunion	Teams understand each other, introduce the rules of the game, and individuals introduce their personality orientation alone	Monday	10-min self-introduction 10-min rule explanation 60-min activity drill
2	Rejection of fear	Role play, release inner pressure, peers encourage each other, self-talk, reflect on anxiety		
3	Give oneself confidence	Understand the factors hindering learning, express English learning feelings, and encourage and help each other in time	Tuesday	10 min to review previous content 30-min rule explanation 90-min activity drill
4	Dare to challenge yourself	Dredge doubts, setbacks and negative evaluations, and learn to change a positive attitude to face problems	Wednesday	
5	Release and evaluation	Look for negative evaluation confusion and learn to transpose thinking	Thursday	
6	Goals and plans	Discuss and plan your own goals, release learning fears, and overcome anxiety and language output barriers	Friday	
7	Sudden training	Cultivate a calm awareness of sudden events, break through yourself, and find your own shining points	Saturday	
8	Expectations for the future	Stimulate the team’s desire to express, review and summarize, and face learning and life optimistically	Sunday	30-min rule explanation 30-min activity drill 30-min summary and evaluation

### Changes in the Overall Level of Academic Pressure and Anxiety Before and After the English Psychological Script Activity Experiment

In order to verify the improvement effect of English psychological script on academic pressure and anxiety, the score difference between *t*-test is used to compare, the analysis results are shown in Table 4. Before the experiment, academic pressure (*M* = 3.61), interpersonal pressure (*M* = 3.54), self-development pressure (*M* = 3.63), social anxiety (*M* = 3.67), evaluation anxiety (*M* = 3.75), examination anxiety (*M* = 3.82), and after the experiment, academic pressure (*M* = 3.17), interpersonal pressure (*M* = 3.14), self-development pressure (*M* = 3.26), social anxiety (*M* = 3.22), evaluation anxiety (*M* = 2.69), and examination anxiety (*M* = 3.15). Therefore, the differences in the academic pressure (*t* = 3.411, *p* < 0.05), interpersonal pressure (*t* = 4.305, *p* < 0.05), self-development pressure (*t* = 2.114, *p* < 0.05), social anxiety (*t* = 2.295, *p* < 0.05), evaluation anxiety (*t* = 3.491, *p* < 0.05) and test anxiety (*t* = 3.145, *p* < 0.05) of college students before and after the English psychological script activity experiment are statistically significant. Therefore, hypothesis H1 is confirmed.

**Table 4:** Difference analysis of overall psychological pressure and anxiety before and after the experiment.

Variable		Before experiment M + SD	After the experiment M + SD	*t*	*p*
Psychological pressure	Academic pressure	3.61 ± 0.43	3.17 ± 0.57	3.411	0.001
	Interpersonal pressure	3.54 ± 0.57	3.14 ± 0.65	4.305	0.001
	Self-development pressure	3.63 ± 0.36	3.26 ± 0.59	2.114	0.029
Anxiety	Social anxiety	3.67 ± 0.46	3.22 ± 1.03	2.295	0.026
	Evaluation anxiety	3.75 ± 1.07	2.69 ± 0.44	3.491	0.001
	Examination anxiety	3.82 ± 0.77	3.15 ± 1.29	3.145	0.003

### Differences in Academic Pressure and Anxiety About Gender Factors Before and After the English Psychological Script Activity Experiment

The improvement effect of English psychological scripts on males and females is also compared by the score difference between *t*-tests. The analysis results are shown in Table 5: Males had academic pressure (*M* = 3.55), interpersonal pressure (*M* = 3.53), self-development pressure (*M* = 3.44), social anxiety (*M* = 3.47), evaluation anxiety (*M* = 3.61), examination anxiety (*M* = 3.55) before the experiment. After the experiment, academic pressure (*M* = 3.22), interpersonal pressure (*M* = 3.09), self-development pressure (*M* = 3.03), social anxiety (*M* = 3.10), evaluation anxiety (*M* = 3.06), and test anxiety (*M* = 3.14). Therefore, the differences in the male college students’ academic pressure (*t* = 3.362, *p* < 0.05), interpersonal pressure (*t* = 4.112, *p* < 0.05), self-development pressure (*t* = 2.111, *p* < 0.05), social anxiety (*t* = 2.287, *p* < 0.05), evaluation anxiety (*t* = 3.421, *p* < 0.05) and examination anxiety (*t* = 3.135, *p* < 0.05) before and after the English psychological script activity experiment are statistically significant.

**Table 5:** Difference analysis of psychological pressure and anxiety between males and females before and after the experiment.

Variable		Before experiment M + SD	After the experiment M + SD	*t*	*p*
Psychological pressure	Academic pressure	3.55 ± 0.44	3.22 ± 0.71	3.362	0.004
	Interpersonal pressure	3.53 ± 0.48	3.09 ± 0.66	4.112	0.001
	Self-development pressure	3.44 ± 0.36	3.03 ± 0.94	2.111	0.043
	Social anxiety	3.47 ± 0.31	3.10 ± 1.03	2.287	0.031
Anxiety	Evaluation anxiety	3.61 ± 1.03	3.06 ± 0.35	3.421	0.002
	Examination anxiety	3.55 ± 0.66	3.14 ± 0.93	3.135	0.004
	Academic pressure	3.58 ± 0.41	3.16 ± 0.78	3.358	0.001
Psychological pressure	Interpersonal pressure	3.64 ± 0.33	3.12 ± 0.61	4.246	0.021
	Self-development pressure	3.58 ± 0.44	3.06 ± 0.93	3.133	0.035
	Social anxiety	3.55 ± 0.37	3.13 ± 0.66	3.262	0.004
Anxiety	Evaluation anxiety	3.60 ± 0.97	3.03 ± 0.33	3.475	0.035
	Examination anxiety	3.70 ± 0.71	3.11 ± 0.41	3.231	0.006

The females’ academic pressure (*M* = 3.58), interpersonal pressure (*M* = 3.64), self-development pressure (*M* = 3.58), social anxiety (*M* = 3.55), and evaluation anxiety (*M* = 3.60), examination anxiety (*M* = 3.70) before the experiment. And the academic pressure (*M* = 3.16), interpersonal pressure (*M* = 3.12), self-development pressure (*M* = 3.06), social anxiety (*M* = 3.13), evaluation anxiety (*M* = 3.03), examination anxiety (*M* = 3.11) after the experiment. Therefore, the differences in academic pressure (*t* = 3.358, *p*<0.05), interpersonal pressure (*t* = 4.246, *p*<0.05), self-development pressure (*t* = 3.133, *p*<0.05), social anxiety (*t* = 3.262, *p* < 0.05), evaluation anxiety (*t* = 3.475, *p* < 0.05), and examination anxiety (*t* = 3.231, *p* < 0.05) of the female college students before and after the English psychological script activity experiment were statistically significant. The hypothesis of H2 can be confirmed by the research results of males and females.

### The Difference in the Academic Pressure and Anxiety of Students of Different Majors Before and After the Experiment of English Psychological Script Activity

The improvement effect of English psychological scripts on students majoring in science and engineering, and humanities and social sciences is also compared by the score difference between the *t*-test. The analysis results are shown in Table 6: before the experiment, the academic pressure (*M* = 3.43), interpersonal pressure (*M* = 3.56), self-development pressure (*M* = 3.44), social anxiety (*M* = 3.49), evaluation anxiety (*M* = 3.65), examination anxiety (*M* = 3.50), and after the experiment, the academic pressure (*M* = 3.07), interpersonal pressure (*M* = 3.04), self-development pressure (*M* = 3.15) and social anxiety (*M* = 3.06), evaluation anxiety (*M* = 3.23), examination anxiety (*M* = 3.11). Therefore, the differences in the academic pressure (*t* = 3.152, *p* < 0.05), interpersonal pressure (*t* = 3.102, *p* < 0.05), self-development pressure (*t* = 2.152, *p* < 0.05), social anxiety (*t* = 2.235, *p* < 0.05), evaluation anxiety (*t* = 3.322, *p* < 0.05), and examination anxiety (*t* = 3.130, *p* < 0.05) of college students majoring in science and engineering before and after the English psychological script activity experiment are statistically significant.

**Table 6:** Difference analysis of psychological pressure and anxiety of students majoring in science and engineering and humanities and social sciences before and after the experiment.

Variable			Before experiment M + SD	After the experiment M + SD	*t*	*p*
Science and engineering	Psychological pressure	Academic pressure	3.43 ± 0.41	3.07 ± 0.59	3.152	0.012
		Interpersonal pressure	3.56 ± 0.46	3.04 ± 0.49	3.102	0.016
		Self-development pressure	3.44 ± 0.56	3.15 ± 0.73	2.152	0.032
	Anxiety	Social anxiety	3.49 ± 0.40	3.06 ± 0.95	2.235	0.026
		Evaluation anxiety	3.65 ± 0.83	3.23 ± 0.64	3.322	0.002
		Examination anxiety	3.50 ± 0.71	3.11 ± 0.46	3.130	0.004
Humanities and Social Sciences	Psychological pressure	Academic pressure	3.63 ± 0.41	3.07 ± 0.75	3.373	0.003
		Interpersonal pressure	3.74 ± 0.30	3.13 ± 0.56	4.261	0.016
		Self-development pressure	3.57 ± 0.29	3.06 ± 0.88	3.242	0.008
	Anxious	Social anxiety	3.53 ± 0.36	3.11 ± 0.53	3.262	0.006
		Evaluation anxiety	3.73 ± 0.76	3.09 ± 0.85	3.436	0.013
		Examination anxiety	3.81 ± 0.85	3.10 ± 0.43	3.250	0.001

Students majoring in humanities and social sciences had academic pressure (*M* = 3.63), interpersonal pressure (*M* = 3.74), self-development pressure (*M* = 3.57), social anxiety (*M* = 3.53), evaluation anxiety (*M* = 3.73), and examination anxiety (*M* = 3.81) before the experiment. After the experiment, academic pressure (*M* = 3.07), interpersonal pressure (*M* = 3.13), self-development pressure (*M* = 3.06), social anxiety (*M* = 3.11), evaluation anxiety (*M* = 3.09), test anxiety (*M* = 3.10). The results are shown in Table 6: the differences in the academic pressure (*t* = 3.373, *p*<0.05), interpersonal pressure (*t* = 4.261, *p*<0.05), self-development pressure (*t* = 3.242, *p*<0.05), social anxiety (*t* = 3.262, *p*<0.05), evaluation anxiety (*t* = 3.436, *p*<0.05), test anxiety (*t* = 3.250, *p*<0.05) of college students majoring in humanities and social sciences before and after the English psychological script activity experiment are statistically significant. The above research results prove that H3 hypothesis is valid.

## Conclusion

In this study, 279 senior students from two universities in Guangdong Province, China were selected as the research objects, and English script activities were used to explore how to improve college students’ psychological pressure and anxiety in life. The results show that: First, verify the research question one “What is the effect of psychological script activity on college students’ psychological stress and anxiety before and after the experiment?” Consistent with the research hypothesis H1, “There is a significant difference in the psychological stress and anxiety of college students before and after the psychological script activity experiment,” before the experiment, the overall psychological pressure and anxiety of college students are at a medium high level. In this range, the order from high to low is examination anxiety, evaluation anxiety, social anxiety, self-development pressure, academic pressure and interpersonal pressure, indicating that college students are suffering from high psychological pressure and anxiety. After the English psychological script experiment, the improvement effect has an obvious effect, reducing anxiety and stress to the medium level. Within this range, the order from high to low is self-development pressure, social anxiety, academic pressure, test anxiety, interpersonal pressure, and evaluation anxiety. From the ranking before and after the experiment, it can also be seen that English psychological script activities have the greatest impact on evaluation anxiety and test anxiety.

Second, verify the research hypothesis H2 of the second research question “What is the effect of psychological script activities on gender and professional categories?” “There are significant differences in the psychological pressure and anxiety of male students and female students before and after the psychological script activity experiment,” it can be seen from the research results, before the experiment, the psychological stress and anxiety of boys and girls were also in a medium high state, and the average of each dimension of psychological stress and anxiety of girls was higher than that of boys. From high to low, the psychological pressure and anxiety of boys are evaluation anxiety, academic pressure, examination anxiety, interpersonal pressure, social anxiety and self-development pressure, while those of girls are examination anxiety, interpersonal pressure, evaluation anxiety, self-development pressure, academic pressure and social anxiety. After the experiment, the improvement of psychological stress and anxiety of boys and girls is in a significant influence state, falling to the medium level. Within this range, the ranking of psychological stress and anxiety of boys from large to small is academic pressure, test anxiety, social anxiety, interpersonal pressure, evaluation anxiety and self-development pressure, while girls are academic pressure, social anxiety, interpersonal pressure, test anxiety, self-development pressure, and evaluation anxiety. From this result, it can be seen that the English script activity has the greatest effect on improving the evaluation anxiety of boys, and the greatest effect on improving the test anxiety and evaluation anxiety of girls.

Finally, the research hypothesis H3 of the second research question is that “there are significant differences in the psychological pressure and anxiety of science and engineering majors and humanities and social science majors before and after the experiment of psychological script activities.” The analysis of the results show that, from the perspective of students majoring in science and engineering, and humanities and social Sciences, before the experiment, the psychological pressure and anxiety of students majoring in humanities and social sciences were higher than those of students majoring in science and engineering, and they are at a medium to high level. From high to low, the psychological pressure and anxiety of students majoring in humanities and social sciences are test anxiety, interpersonal pressure, evaluation anxiety, academic pressure, self-development pressure and social anxiety, while those of students majoring in science are evaluation anxiety, interpersonal pressure, test anxiety, social anxiety, self-development pressure, and academic pressure. After the experiment, the improvement effect of students majoring in humanities and social sciences and students majoring in science and technology also decreased significantly to the medium level. Within this range, the order of humanities and social sciences from high to low is interpersonal pressure, social anxiety, examination anxiety, evaluation anxiety, academic pressure and self-development pressure, while science and engineering is evaluation anxiety, self-development pressure, examination anxiety, academic pressure, social anxiety and interpersonal pressure. This analysis shows that English script activities have an average impact on the psychological pressure and anxiety of students majoring in humanities and social sciences, while they have the highest impact on the interpersonal pressure of students majoring in science and technology.

In summary, we can see from the above research that whether it is from professional differences or gender differences, English psychological scripts have a positive effect on the improvement of psychological pressure and anxiety. Especially in the gender observation, most of the previous studies have revealed that the stress and anxiety levels of females and males are different, and there are also many studies that describe the stress of females is higher than that of males. The results of this study show that psychodrama activities can not only reduce the stress and anxiety of males and females, but also add and use different link components for different genders to improve the level of mental health. For example, if the participants are males or females with high evaluation anxiety or examination anxiety, they can carry out the activities of “rejection of fear” and “releasing and evaluation” in psychological drama activities, and actively guide them to deal with their own psychological evaluation problems. Other phenomena can also be alleviated by imitating the example, and customized additions can be made to realize the refinement of the right way to intervene in the object’s mental health problems. From the data before the experiment, we can also know that today’s college students are facing a high level of pressure and negative anxiety experience on campus. Properly guiding them to reduce pressure and reduce burden can improve their living conditions on a benign campus. Generally, senior students are facing the pressure of learning and employment, which is the goal we need to pay more attention to. In the English script, there is a theme to solve the negative emotion and psychological pressure, which can appropriately reduce the psychological burden of students. If other links are developed in the English script, it may be better to try to integrate English songs, musicals, and other components. Therefore, in future research, suggestions in this regard can be considered, not only observe the best mental health intervention effect of each link on the object in the link time allocation, for example, whether there are other better creative links for females to enrich the participation and entertainment of psychodrama, so that psychodrama has more significance for people’s psychological treatment, We can also try to use other languages to carry out psychodrama therapy, observe the improvement effect of college students in activities, and bring more new significance to college students’ mental health and education, so as to bring more new significance to college students’ mental health and education.

## Data Availability Statement

The original contributions presented in the study are included in the article/supplementary material, further inquiries can be directed to the corresponding author.

## Ethics Statement

Ethical review and approval was not required for the study on human participants in accordance with the local legislation and institutional requirements. Written informed consent from the patients/participants was not required to participate in this study in accordance with the national legislation and the institutional requirements.

## Author Contributions

LM was responsible to the research design, searched the literature, analyzed the data and contributed to the writing of the paper. BD and XH contributed to the collection and collation of the data and the writing of the paper. All authors contributed to the article and approved the submitted version.

## Conflict of Interest

The authors declare that the research was conducted in the absence of any commercial or financial relationships that could be construed as a potential conflict of interest.

## Publisher’s Note

All claims expressed in this article are solely those of the authors and do not necessarily represent those of their affiliated organizations, or those of the publisher, the editors and the reviewers. Any product that may be evaluated in this article, or claim that may be made by its manufacturer, is not guaranteed or endorsed by the publisher.
